# Identification of the complete mitochondrial genome of *Monomia gladiator* (Decapoda: Brachyura: Portunidae) and its phylogenetic relationship

**DOI:** 10.1080/23802359.2018.1437827

**Published:** 2018-02-10

**Authors:** Zhuofang Xie, Hanafiah Fazhan, Xincang Li, Huaqiang Tan, Khor Waiho, Mengyun Guan, Fan Lin, Hongyu Ma

**Affiliations:** a Guangdong Provincial Key Laboratory of Marine Biotechnology, Shantou University, Shantou, China;; b East China Sea Fisheries Research Institute, Chinese Academy of Fishery Sciences, Shanghai, China

**Keywords:** Monomia gladiator, mitochondrial genome, phylogeny

## Abstract

The complete mitochondrial genome sequence plays an important role in phylogenetic studies. In this study, the complete mitochondrial genome of *Monomia gladiator* was obtained by Illumina and Sanger sequencing techniques. The circular genome was 15,878 bp in length, consisting of 13 protein-coding genes, 22 transfer RNA genes, 2 ribosomal RNA genes, and a putative control region. This whole mitogenome composition was 33.32% for A, 35.69% for T, 11.75% for G, and 19.24% for C, respectively. The phylogenetic analysis suggested that *M. gladiator* was genetically closest to three Portunidae species (*Charybdis japonica*, *C. feriata* and *Thalamita crenata*). The newly described mitogenome may facilitate the phylogenetic studies on Portunidae crabs.

The gladiator swimming crab, *Monomia gladiator* was an important crab species under the family Portunidae with economic potential. It is mainly distributed in Japan, Indonesia, Thailand, India, New Zealand, Australia, and China (Zhang 1997). The genus *Monomia* has been considered as a complex species with confused nomenclature and taxonomy for a long time (Koch et al. 2017). Recently, molecular studies based on *16S* mitochondrial gene and histone H3 fragment indicated that Portunoidea includes morphologically diverse taxa (Schubart and Reuschel 2009). Complete mitochondrial genome will facilitate understanding the phylogeny and species evolution in depth (Yang et al. 2017). However, there are only COI (Spiridonov et al. 2014) and partial 16S rRNA sequences (Koch et al. 2017) of *M. gladiator* available in the Genbank database. In this study, we firstly reported the complete mitochondrial genome of *M. gladiator* and analyzed its phylogenetic position.

Specimens of *M. gladiator* were collected from fish market in Xiamen City (24.463015°N, 118.087908°E), China and stored in Marine Biology Institute of Shantou University. Total genomic DNA was extracted from the muscle tissue using the described method (Ma et al. 2009). The complete mitogenome sequence was obtained by Illumina and Sanger sequencing methods.

The complete mitochondrial genome of *M. gladiator* was 15,878 bp in length (GenBank accession number: MG770549), and consisted of 13 protein-coding genes, 22 transfer RNA genes, 2 ribosomal RNA genes, and a putative control region. The nucleotide composition was 33.32% for A, 35.69% for T, 11.75% for G, and 19.24% for C, respectively. Of the 37 genes, 23 were encoded by the heavy strand, while 14 were encoded by the light strand. The arrangement and composition of the mitochondrial genome were similar to those of *Charybdis feriata* (Ma et al. 2015) and *Thalamita crenata* (Tan et al. 2016). The length of the 13 protein-coding genes ranged from 162 bp (*ATP8*) to 1728 bp (*ND5*). The large ribosomal RNA (16S) and small ribosomal RNA (12S) were 1352 bp and 874 bp in length, respectively. The tRNA^Val^ was sandwiched between these two ribosomal RNA genes.

In order to determine the phylogenetic position of *M. gladiator,* a ML tree was constructed using the concatenated sequences of 12 protein-coding genes (except *ND6*) (Figure 1). *Hapiosquilla harpax* was used as an outgroup for tree rooting. The result showed that *M. gladiator* was clustered together with three Portunidae species (*C. japonica, C. feriata, and T. crenata*), indicating that it was genetically closest to these three species. This result was also confirmed by a previous study (Spiridonov et al. 2014), in which *M. gladiator* was suggested to have closer genetic relationship to genera *Charybdis* and *Thalamita* than *Portunus* based on *histone H3*, *COI*, and *28S* genes. The complete mitochondrial genome of *M. gladiator* could provide valuable information for the phylogenetic relationship of Portunidae.

**Figure 1.**
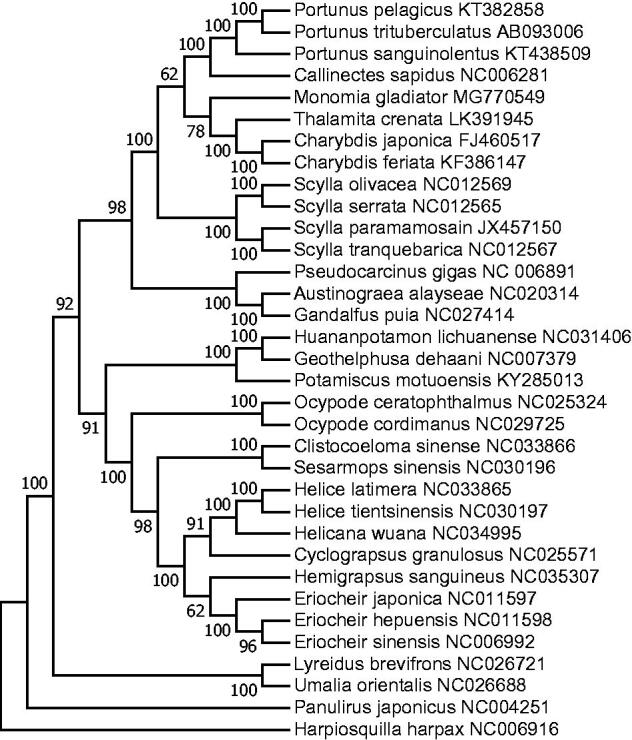
Molecular phylogeny of *M. gladiator* and other related species constructed using 12 protein-coding genes of mitochondrial genome.
